# The Role of Visfatin/NAMPT in the Pathogenesis of Psoriasis

**DOI:** 10.3390/metabo15090590

**Published:** 2025-09-06

**Authors:** Mateusz Matwiejuk, Agnieszka Kulczyńska-Przybik, Bartłomiej Łukaszuk, Hanna Myśliwiec, Piotr Myśliwiec, Adrian Chabowski, Barbara Mroczko, Iwona Flisiak

**Affiliations:** 1 Department of Dermatology and Venereology, Medical University of Bialystok, Zurawia 14, 15-540 Bialystok, Poland; hanna.mysliwiec@gmail.com (H.M.);; 2 Department of Neurodegeneration Diagnostics, Medical University of Bialystok, 15-269 Bialystok, Poland; 3 Department of Physiology, Medical University of Bialystok, 15-222 Bialystok, Poland; 4 1st Clinical Department of General and Endocrine Surgery, Medical University of Bialystok, 15-276 Bialystok, Poland

**Keywords:** psoriasis, skin diseases, protein, visfatin, NAMPT, HDL-C

## Abstract

Introdcution: Psoriasis is a complex, chronic, immunologically, inflammatory, and environmentally mediated disease which may affect not only the skin, but also nails, and joints. This dermatosis is known for hyperproliferation, parakeratosis, dysregulated differentiation of keratinocytes, lack of granular layer of the skin and impaired apoptosis of keratinocytes. Methods Fifty patients with psoriasis and twenty-eight healthy individuals were enrolled in the study. Serum samples were collected both from the psoriatic patients and patients with an inguinal hernia, who served as the control group. Visfatin levels were measured by enzyme-linked immunosorbent assay. Various proteins have been well described as key contributors to the complex pathogenesis of psoriasis. Results: In our study, we found that serum visfatin levels were significantly higher in the psoriatic group compared to the control group. Interestingly, we observed a positive and statistically significant correlation between serum visfatin levels and HDL-C concentrations in patients with psoriasis. Discussion: An elevated HDL-C level in psoriatic serum might be a sign of a compensatory response to systemic inflammation, or a marker of metabolic dysfunction, or an early prognostic signal in disease progression. However, no significant correlations were found between visfatin levels and the Psoriasis Area and Severity Index (PASI) score. Conclusions: In summary, our findings indicate that visfatin levels are significantly altered in the serum of patients with psoriasis compared to the control group and it could be a pivotal point of understanding the pathogenesis of psoriasis and a new way of implementing therapeutic procedures.

## 1. Introduction

Psoriasis is a common chronic inflammatory and autoimmune skin disease characterised by alternating periods of exacerbation and remission [1]. The development and flares of psoriasis are often driven by the interplay between genetic predisposition and environmental triggers [2]. It has been estimated that roughly 125 million people worldwide suffer from the condition. The number reflects a significant burden imposed by the dermatitis on the public health system [3]. Data provided by the World Health Organisation revealed that the prevalence of psoriasis differs with respect to a particular population and ranges from mere 0.09% to a significant 11.43% [4]. According to another source, several high-income regions, like Australasia (1.99% (0.64–6.60%)), Western Europe (1.92% (1.07–3.46%)), Central Europe (1.83% (0.62–5.32%)), North America (1.50% (0.63–3.60%)), and high-income Southern Latin America (1.10% (0.36–2.96%) show higher prevalence of psoriasis than the rest of the world. The incidence rate per 100,000 person-years (95% CI) [5]. Intriguingly, a greater percentage of psoriasis is observed in males than in females; however, earlier onset of psoriatic symptoms in seen in women. Moreover, the bimodal distribution of its onset is usually observed, with the peaks of psoriasis incidence found in the patients in their 30s and 60s [6]. The co-occurrence of psoriasis with other medical conditions can indeed amplify its negative impact on the overall health of an individual. The most common comorbidities of psoriasis are obesity [7], hypertension [8], type 2 diabetes [9], non-alcoholic fatty liver disease [10], cardiovascular events [11], and metabolic syndrome (MetS) [12]. According to Wu et al. [13], the economic burden of comorbidities associated with psoriasis was estimated at approximately $36.4 billion in 2013, based on data from the United States alone [13].

Psoriasis may appear in various clinical forms including plaque, guttate, flexural, pustular, and erythrodermic psoriasis. The most common type is plaque psoriasis, characterised by well-demarcated pink to red plaques covered with fine or thick white-silvery scale. Additionally, this dermatosis typically exhibits a fairly symmetrical distribution and most commonly affects the scalp, trunk, and extensor surfaces of the elbows and knees [14]. Psoriasis is a dermatosis with a complex pathogenesis. One of the contributing factors is believed to be the abnormal expression of regulatory and structural proteins [15]. It has also been reported that alarmins, antimicrobial peptides, autoantigens, cytokines, growth factors, and proteases play crucial roles in the development and progression of psoriasis [16]. Moreover, dendritic cells that normally circulate in the body end up settling in the epidermis, they become strongly activated in the specific areas affected by psoriasis. As a result, they expel interferon α (IFN-α), tumour necrosis factor α (TNF-α), interleukins (IL-20 and IL-23), inducible nitric oxide synthase, and lymphoid-organising chemokines (CCL19, CCL21, CXCL12, and CCL18) [17].

Recent studies revealed several proteins that could be key players in the pathogenesis and progression of psoriasis, such as visfatin/nicotinamide phosphoribosyltransferase (NAMPT) [18,19]. It is also known as a NAMPT, which is an essential enzyme in the NAD+ biosynthesis pathway, where it is responsible for catalysing the transformation of nicotinamide to nicotinamide mononucleotide [20].

Multiple cell types manufacture visfatin, including adipocytes [20], neurons [21], cancer cells [22,23], and immune cells [24].

Visfatin plays a key role in regulating different tumour-related processes, like apoptosis, proliferation, migration, angiogenesis, and invasion [25,26].

Moreover, visfatin is a proinflammatory protein, which enhances the production and secretion of proinflammatory cytokines, such as the following: interleukin-6, TNF-α, and interleukin-1 beta in human monocytes and endothelial cells [27]. In addition to its above-mentioned proinflammatory role, visfatin also boosts the expression of co-stimulatory molecules (CD80, CD40, and ICAM-1) on monocytes, which further increases T-cell activation through signalling pathways involving p38 and MEK [28]. In keratinocytes, it promotes the secretion of CXCL8 (IL-8), CXCL10 (IP-10), CCL20 (MIP-3α)), in response to TNF-α stimulation [29]. Apart from this, visfatin intensifies the expression of antimicrobial peptides, including cathelicidin antimicrobial peptide (CAMP), human beta-defensins 2 and 3 (hBD-2, hBD-3), and S100A7 [30].

Visfatin also augments the production of vascular endothelial growth factor (VEGF) and matrix metalloproteinases (MMP-2 and MMP-9), which are essential key players in angiogenesis [31].

Despite the advancements above, visfatin has been identified as a potential player in the pathogenesis of psoriasis. However, its precise role and the link between its serum levels and disease severity still remain elusive.

This article aims to deepen our understanding of the role of visfatin in the development and progression of psoriasis. We assessed the concentration of visfatin in patients with psoriasis compared to a healthy control group and evaluated its association with Psoriasis Area and Severity Index (PASI) scores (indicating disease severity), as well as with various biochemical and clinical parameters.

## 2. Materials and Methods

A total of 50 patients (20 females and 30 males) with active plaque-type psoriasis, at a median age of 51.0, who were hospitalised in the clinic of Dermatology and Venereology of the Medical University of Bialystok, and 28 controls, suffering from inguinal hernia (24 females and 4 males) at a median age of 42.0, who were hospitalised in the I Clinic of General and Endocrine Surgery, Medical University of Bialystok, were enrolled in this study. The severity of psoriasis was estimated using the Psoriasis Area and Severity Index (PASI) [32]. Body mass index (BMI) was calculated based on self-reported weight and height [33]. None of the patients or controls were under dietary restriction. History of chronic diseases like hypertension, liver disease (e.g., non-alcoholic fatty liver disease, cardiovascular disease, diabetes mellitus), and results of the laboratory tests were collected from hospital records of the patients, and these patients were excluded from the study. Laboratory tests were performed before the treatment. All psoriatic and healthy patients signed their written informed consent before enrolment in this study. The research protocol was approved by the local university bioethical committee (no APK.002.272.2025) and followed the principles of the Helsinki Declaration. Peripheral blood samples were collected after an overnight fast and prior to the initiation of treatment. After centrifugation, the serum was stored at −80 °C until it was further analysed.

### 2.1. Visfatin Analysis

The concentration of visfatin (ng/mL) in the serum was assessed by the commercially available kit Human Visfatin (NAMPT) ELISE, BioVendor group enzyme immunoassay kit (Brno, Czech Republic) on Biotek Synergy 2 Plate Reader Multi-Mode using Gen5™ 2.0 Data Analysis Software (version 2.01.14). The quantitative determination of the tested protein was performed according to the manufacturer’s procedure in the Department of Neurodegeneration Diagnostics. The serum samples were undiluted. Standards and samples were run in duplicates with a coefficient of variance (CV) < 20%. Absorbance was read at 450 nm.

### 2.2. Statistical Analysis

Statistical analysis was conducted using Julia ver. 1.10.8 (https://julialang.org/, accessed on 2 June 2025). Categorical variables (Table 1) were presented as counts and were compared using Fisher’s Exact test for count data. The between group comparisons for continuous variables (Table 1) were made with Student’s *t*-test or Mann–Whitney *U* test (also known as Wilcoxon test). The choice of the test was made based on the fulfilment of normality (assessed with Shapiro–Wilk’s test) and variance homogeneity (estimated with Fligner-Killeen test) criteria. The correlation analysis (heatmaps) were constructed based on the Pearson’s correlation coefficients. The obtained *p*-values for correlations were adjusted for multiple comparisons (Benjamini–Hochberg correction). A set of selected statistically significant correlations was presented on scatterplots with a trend line (obtained from linear regression) overlaid on them. Multiple logistic regression (Table 2) was conducted to obtain the so called minimal adequate model. The obtained *p*-values < 0.05 were deemed to be statistically significant.

## 3. Results

### 3.1. Study Population

In this study, 50 patients (20 females and 30 males) with active plaque-type psoriasis and 28 healthy patients (24 females and 4 males) were analysed. The median age in the control group was 42, ranging from 37.5 to 47.2 years; the median age in the psoriatic group was 51, ranging from 34.2 to 66.0 years. The average duration of psoriasis was 16 years. In the control group, the median body mass was 69.5 kg (63.8–79.2) kg, the mean height was 165.0 (161.5–170.2) cm, and the median BMI was 25.1 (23.5–27.9) kg/m^2^. Approximately half patients from the control group (n = 12) (43%) had a normal weight, 11 (39%) were overweight, and 5 (18%) suffered from obesity. In the psoriasis group, the median body mass was 84.0 (75.5–95.8) (kg), the mean height was 172.5 (164.2–176.0) (cm), and the median BMI was 29.0 (23.9–31.8). A total of 19 patients dealing with psoriasis suffered from obesity (38%), 17 (34%) were overweight, and 14 (28%) had a normal weight. The examined group, 7 (14%) patients had a mild (psoriasis area and severity index (PASI < 10)) form of psoriasis, 43 psoriatic patients (86%) were dealing with moderate to severe psoriasis (PASI > 10), 26 patients (52%) had PASI 10–20, and 17 patients (34%) had a PASI > 20.

In the control group, we included patients who were planned to undergo surgery due to inguinal hernia. The patients did not suffer from any metabolic and skin diseases. In the study group, we included adult patients dealing with plaque psoriasis with at least a 4-week gap without systemic treatment (methotrexate, acitretin, or cyclosporine). Based on that, we excluded psoriatic patients who were on systemic anti-psoriatic treatment. Data on systemic diseases were collected from patients’ hospital records: hypertension, liver diseases (e.g., non-alcoholic fatty liver disease (NAFLD)), heart diseases, and diabetes mellitus. None of the patients in the study or control group were subject to dietary restrictions, did not use protein-rich products during the study, and did not take any medications that could affect protein metabolism.

Table 1 summarises the main clinical features of the psoriatic group and the control group.

### 3.2. Visfatin Parameter

**Figure 1:**
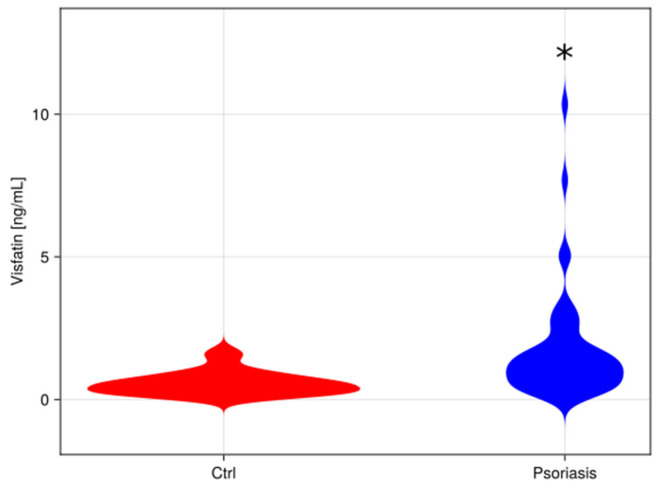
Comparison between visfatin levels in control patient’s serum, and psoriatic patient’s serum [ng/mL]. A violin plot comparing data distributions in both groups. *—different vs. control group (*p* < 0.05).

The median concentration of visfatin found in the serum of psoriatic individuals (1.2 ng/mL) was found to be significantly higher (*p* < 0.05) than in the serum of healthy patients (0.5 ng/mL). Figure 1 shows different visfatin levels in healthy patients and psoriatic patients.

## 4. Discussion

### The Role of Visfatin

In this study, we investigated serum visfatin levels in patients with psoriasis and compared them to the levels in healthy individuals. Moreover, we also assessed the relationship between visfatin levels and various clinical and laboratory parameters within the psoriatic patient group.

Our findings showed that the level of visfatin was significantly higher in the serum of patients suffering from psoriasis compared with serum samples of the control group. (Figure 1). Moreover, logistic regression analysis demonstrated that visfatin levels may serve as a prognostic factor for the condition (Table 2). Furthermore, our study highlighted a statistically significant positive correlation between visfatin and HDL-C in the serum of the examined group (r = 0.41, *p* < 0.05).

Mercurio et al. [34] highlighted the crucial role of visfatin/NAMPT-mediated NAD+ metabolism in fuelling the immune responses observed in psoriatic skin. Firstly, an intracellular visfatin/NAMPT is significantly upregulated by Th1/Th17-cytokines and directly impacts keratinocytes by promoting excessive proliferation and hindering their normal differentiation. Secondly, the increase in NAD+ levels due to visfatin/NAMPT activity works in conjunction with psoriasis-related cytokines to enhance the production of inflammatory chemokines, which are essential for attracting neutrophils and Th1/Th17 cells to the affected area. Thirdly, an extracellular visfatin/NAMPT, released in large quantities by keratinocytes and dermal fibroblasts, exerts paracrine effects on endothelial cells. It stimulates their proliferation and migration towards the inflamed skin and also increases the expression of ICAM-1 (a cell adhesion molecule) and other chemokines, facilitating the recruitment of leukocytes into the inflamed skin. In summary, the visfatin/NAMPT-mediated NAD+ salvage pathway plays a significant role in the development of psoriasis by intensifying the auto-inflammatory responses within the epithelial cells [34].

Similarly, Martinez-Morcillo et al. [35] further supported the role of the visfatin/NAMPT-mediated pathway in psoriasis by demonstrating that inhibiting the NADPH oxidases/NAMPT/PARP/apoptosis-inducing factor mitochondria-associated 1 (AIFM1) axis decreased the expression of pathology-associated genes in human 3D skin models of psoriasis. An abnormal increase in visfatin/NAMPT and poly(ADP-ribose) (PAR) polymerase (PARP) activity, along with the nuclear translocation of AIFM1, was observed in lesional skin from psoriasis patients. The researchers concluded that hyperactivation of PARP1, triggered by reactive oxygen species-induced DNA damage and fuelled by NAD+ derived from visfatin/NAMPT, led to skin inflammation through a form of programmed cell death known as parthanatos [35].

Likewise, Ismail et al. [36] presented in their study that patients with psoriasis exhibited significantly elevated serum levels of visfatin compared to healthy controls. Furthermore, their findings indicated a significant positive correlation between serum visfatin concentrations and both Psoriasis Area and Severity Index (PASI) scores (*p* < 0.01) and disease duration (*p* < 0.01). Consequently, they proposed visfatin as a potential marker for assessing the severity and chronicity of psoriasis [36].

The research of Okan et al. [37] supported the idea that visfatin may be a relevant marker of psoriasis. They found that serum visfatin levels were significantly higher in psoriatic patients compared to healthy controls (*p* = 0.002). Furthermore, they observed a significant correlation between Psoriasis Area and Severity Index (PASI) scores and visfatin levels (*p* = 0.011), similar to the findings of Ismail et al. The above suggests that visfatin levels might reflect the disease severity in psoriasis [37]. In our study, we also found higher levels of visfatin in the serum of patients with psoriasis, but in contrast to their findings, we did not observe a correlation between visfatin levels and Psoriasis Area and Severity Index (PASI) score.

Zou et al. [38] reported that visfatin/NAMPT, is a promising biomarker for the early diagnosis of psoriasis vulgaris due to their high diagnostic value identified through bioinformatical analysis and clinical sample validation. Additionally, the researchers described a potential role of visfatin in the regulation of autophagy, which provides a potential mechanistic link to psoriasis vulgaris development [38].

Similarly to the above-mentioned study, the study by Bai et al. [39] highlighted that visfatin/NAMPT was a key gene potentially influencing psoriasis development through its regulatory role in autophagy [39].

#### The Link Between Visfatin and HDL-C

**Figure 2:**
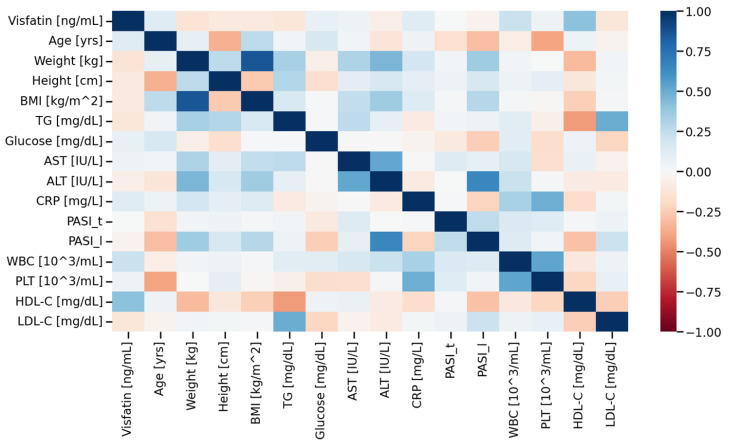
Correlation matrix (heatmap) in the psoriatic group. Pearson correlation coefficients are depicted as the shades of blue (positive correlation) or red (negative correlation). BMI—body mass index, CRP—C-reactive protein, TG—triacylglycerol, AST—Aspartate transaminase, ALT—Alanine transaminase, PASI—Psoriasis Area Severity Index.

**Figure 3:**
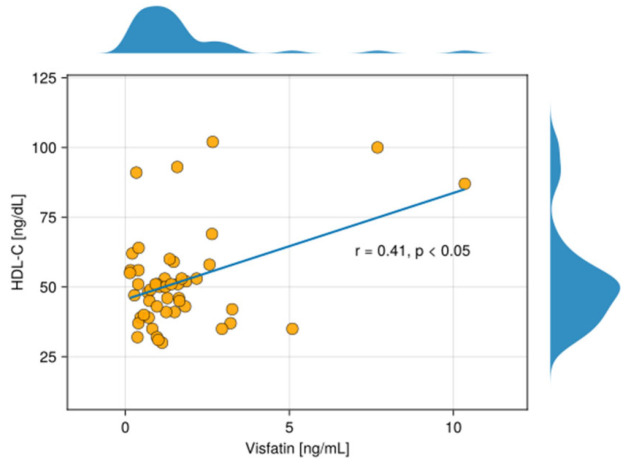
The scatterplot shows a correlation between visfatin and high-density lipoprotein (HDL-C) in the serum of patients with psoriasis. Above and on the right of the scatter plot, density plots are overlaid, depicting the distribution of the variables on the X- and Y-axis, respectively.

In our study, we observed a statistically significant, positive correlation between visfatin levels and HDL-C levels in the serum of the psoriatic group (r = 0.41, *p* < 0.05) (Figure 2 and Figure 3). To the best of our knowledge, this outcome has not been previously reported.

Dagdelen et al. [40] observed that visfatin levels were much more elevated within the patients with psoriasis and MetS, compared to psoriasis alone. This suggests that the presence of MetS significantly impacts the levels of the adipokine, even in the context of psoriasis. However, no link between HDL and visfatin was described by the authors [40]. Brazzelli et al. [41], Chyl-Surdacka et al. [42], and Coban et al. [43] also did not find any correlation between visfatin and HDL-C in the serum of patients dealing with psoriasis [41,42,43].

In our study, visfatin levels were positively correlated with HDL-C concentrations. While this finding may initially appear counterintuitive—given visfatin’s well-established proinflammatory role and HDL-C’s traditionally protective, anti-inflammatory profile—several explanations may account for this association. In chronic inflammatory conditions such as psoriasis, HDL particles can undergo structural and functional modifications, leading to the formation of dysfunctional HDL that retains quantitative presence (measured as HDL-C) but loses its protective properties [44]. Additionally, visfatin may play a role in modulating lipid metabolism, potentially stimulating hepatic HDL production as a compensatory mechanism in response to inflammation. Another possibility is that visfatin and HDL-C are both modulated by shared metabolic or inflammatory pathways, such as those associated with insulin resistance, subclinical metabolic disturbances, or early atherosclerotic changes—even in the absence of clinically diagnosed metabolic syndrome. It should be noted that the observed correlation between visfatin and HDL-C reflects an association rather than a causal relationship. Such associations can be influenced by multiple metabolic and inflammatory factors and therefore must be interpreted with caution. Future longitudinal and mechanistic studies are needed to clarify the direction and clinical significance of this relationship

**Figure 4:**
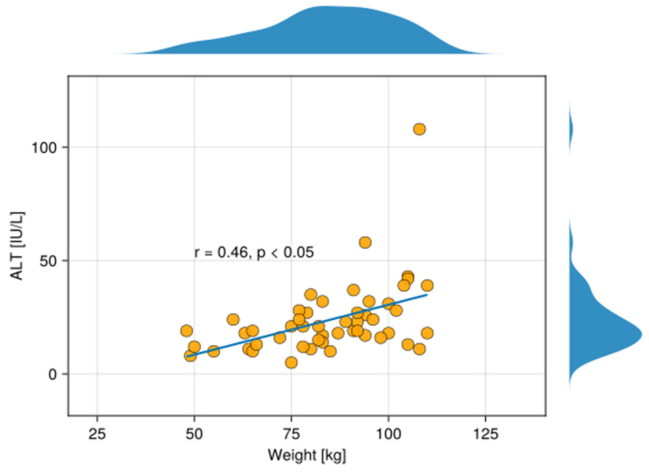
The scatterplot shows a correlation between ALT in the serum and the weight of patients with psoriasis. Above and on the right of the scatter plot, density plots are overlaid, depicting the distribution of the variables on the X- and Y-axis, respectively.

Moreover, in our study, ALT levels were positively correlated with HDL-C weight of psoriatic patients (Figure 4).

Patients with psoriasis are at a significantly higher risk of developing non-alcoholic fatty liver disease (NAFLD) and obesity compared to the general population [45]. Serum ALT levels in psoriatic patients can be elevated due to the association between psoriasis and liver condition, particularly NAFLD and obesity [46]. Lower plasma visfatin levels are associated with impaired hepatic mitochondrial function, which may lead to liver abnormality and an elevated serum ALT level. Furthermore, this points out a possible inverse relationship in certain contexts, where reduced visfatin may reflect compromised liver metabolism, leading to higher ALT levels [47]. Patients dealing with psoriasis and NAFLD, with elevated ALT levels, are vulnerable to having an increased body mass index (BMI). In obese patients, ALT serum levels correlate positively with fat mass index in men (r = 0.23, *p* = 0.004) and lean mass index in both sexes (r = 0.32, *p* < 0.0001 in men; r = 0.13, *p* = 0.031 in women), although the connection with fat mass is not as underlined as in women. Moreover, psoriatic patients with elevated ALT may be at higher cardiovascular risk than women, necessitating aggressive management of obesity and lifestyle factors [46].

However, several limitations of our study should be acknowledged. Although patients with diagnosed and/or treated metabolic diseases were excluded, it remains possible that some individuals had undiagnosed or subclinical metabolic conditions that could have influenced both visfatin and HDL-C levels. Therefore, the observed correlation might reflect an early or pre-diagnostic metabolic state, potentially serving as a prognostic indicator rather than a direct mechanistic link. In addition, we cannot fully exclude the influence of important clinical and demographic confounders such as age, sex, body mass index, comorbidities, or lifestyle factors, which may affect both adipokine and lipid profiles. The relatively small study cohort (50 psoriatic patients and 28 controls) further limits the statistical power and generalisability of the findings. Finally, all blood samples were collected prior to the initiation of psoriasis-specific treatment, and it remains unclear how systemic therapy might subsequently affect the visfatin–HDL-C relationship. Larger, longitudinal studies with multivariable adjustment are warranted to validate these findings and to better elucidate the underlying mechanisms.

## 5. Conclusions

Our findings suggest that elevated visfatin levels in the serum of psoriatic patients, compared to healthy controls, may reflect a broader role of this adipokine in the systemic pathogenesis of psoriasis. Beyond skin manifestations, these results support the notion that psoriasis is associated with underlying metabolic and inflammatory disturbances, implicating circulating proteins such as visfatin in the broader metabolic profile of affected individuals. This observation highlights visfatin’s potential not only as a biomarker of systemic involvement but also as a mediator linking psoriasis to metabolic comorbidities.

Moreover, we identified a statistically significant positive correlation between visfatin and HDL-C levels in psoriatic patients. Although HDL-C is typically considered cardioprotective and anti-inflammatory, its functional quality may be impaired in chronic inflammatory states. The unexpected correlation with HDL-C underscores the need for further research into the functional state of lipoproteins in chronic inflammation and their interaction with adipokines. Importantly, as correlation reflects association rather than causation and may be influenced by multiple confounding factors, this finding should be interpreted with caution and validated in larger, controlled studies.

## Figures and Tables

**Table 1:** Clinical and biochemical characteristics of the control group and psoriatic patients. Data are presented as median and interquartile range. *—different vs. control (*p* < 0.05).

Control	Psoriasis	Clinical and Laboratory Features
42.0 (37.5–47.2)	51.0 (34.2–66.0)	Age [years]
69.50 (63.8–79.2)	84.0 (75.5–95.8) *	Weight [kg]
165.0 (161.5–170.2)	172.5 (164.2–176.0) *	Height [cm]
25.1 (23.5–27.9)	29.0 (23.9–31.8) *	BMI [kg/m^2^]
1.0 (1.0–2.0)	3.2 (1.5–6.9) *	CRP [mg/dL]
86.5 (78.8–91.0)	85.0 (80.0–93.0)	Glucose [mg/dL]
73.0 (67.5–82.0)	116.0 (86.2–134.5)	TG [mg/dL]
17.5 (15.0–21.0)	20.0 (16.2–27.0) *	AST [U/L]
15.5 (11.5–18.2)	19.0 (14.2–27.8) *	ALT [U/L]
24/4	20/30 *	Sex [no. female/no. male]

BMI—body mass index, CRP—C reactive protein, TG—triacylglycerol, AST–Aspartate transaminase, ALT—Alanine transaminase.

**Table 2:** Logistic regression analysis, minimal adequate model. Dependent variable: group (Control = 0, Psoriasis = 1), predictors: Visfatin [ng/mL], Age [yrs], Weight [kg], Height [cm], TG [mg/dL], Glucose [mg/dL], AST [IU/L], ALT [IU/L], CRP [mg/L].

	Coef.	Lower 95%	Upper 95%	*p*-Value
(Intercept)	−5.292	−8.734	−1.85	<0.05 *
Weight [kg]	0.059	0.016	0.103	<0.05 *
Visfatin [ng/mL]	1.555	0.41	2.701	<0.05 *

*—different vs. control (*p* < 0.05).

## Data Availability

Data available upon request from the corresponding author.

## Abbreviations

MetS	metabolic syndrome
IFN-α	interferon α
TNF-α	tumour necrosis factor α
NAMPT	nicotinamide phosphoribosyltransferase
BMI	body mass index
CRP	C-reactive protein
TG	triacylglycerol
AST	Aspartate transaminase
ALT	Alanine transaminase
